# Fatal Case of Crimean-Congo Hemorrhagic Fever Caused by Reassortant Virus, Spain, 2018

**DOI:** 10.3201/eid2704.203462

**Published:** 2021-04

**Authors:** Anabel Negredo, Rafael Sánchez-Arroyo, Francisco Díez-Fuertes, Fernando de Ory, Marco Antonio Budiño, Ana Vázquez, Ángeles Garcinuño, Lourdes Hernández, César de la Hoz González, Almudena Gutiérrez-Arroyo, Carmen Grande, Paz Sánchez-Seco

**Affiliations:** National Center of Microbiology, Madrid, Spain (A. Negredo, F. Díez-Fuertes, F. De Ory, A. Vázquez, L. Hernández, P. Sánchez-Seco);; Complejo Asistencial de Ávila, Ávila, Spain (R. Sánchez-Arroyo, M.A. Budiño, A. Garcinuño, C. de la Hoz González, C. Grande);; CIBERESP, Madrid (A. Vázquez); La Paz Hospital, Madrid (A. Gutiérrez-Arroyo)

**Keywords:** Crimean Congo hemorrhagic fever virus, Hyalomma, RT-PCR, molecular epidemiology, phylogenetic analyses, tick-borne illness, vector-borne infections, viruses, reassortant, zoonoses, Spain, ticks

## Abstract

In August 2018, a fatal autochthonous case of Crimean-Congo hemorrhagic fever was confirmed in western Spain. The complete sequence of the viral genome revealed circulation of a new virus because the genotype differs from that of the virus responsible for another case in 2016. Practitioners should be alert to possible new cases.

A fatal case of Crimean-Congo hemorrhagic fever (CCHF) detected in Spain in 2018 was caused by a different genotype, a reassortant virus, than the genotype of a previous case detected in 2016. This unexpected variability contrasts with the situation in other CCHF-endemic countries. Because CCHF is a zoonotic disease and animal migratory routes between Europe and Africa usually pass through Spain, data about genetic sequences are crucial for monitoring infections in humans, developing suitable detection tools, and providing information about the dynamics of virus circulation and spread. 

## The Case

On July 31, 2018, a 74-year-old man sought care at Nuestra Señora de Sonsoles Hospital (Ávila, Spain) with fever (39.2°C), pain in the sacroiliac area, chills, shivering, and a feeling of dizziness without loss of consciousness. No relevant physical findings or analytical parameters were detected (Table 1). While in the hospital, the patient remained stable and in good general condition. He was discharged for observation at home, afebrile, with a diagnosis of febrile syndrome with bacteremia and a prescription of amoxicillin/clavulanic acid and instructions to take acetaminophen if fever redeveloped.

**Table 1:** Serial hematologic and biochemical parameters, vital signs, and treatments administered for Crimean-Congo hemorrhagic fever patient, Spain, 2018*

Variable	Jul 31	Aug 4	Aug 5	Aug 6	Aug 7
Hematologic parameters					
Hemoglobin, g/dL	13.5	14.5	12.9	12.4	9.4
Hematocrit, %	39.4	42.9	37.7	36.4	28
Leukocytes, × 10^−3^ cells/mm^3^	10.7	4.1	3.6	4.6	5.9
Neutrophils, × 10^−3^ cells/mm^3^	9.5	2.7	2.4	2.8	3.6
Lymphocytes, × 10^−3^ cells/mm^3^	0.4	0.9	0.8	1.1	1.3
Platelets, × 10^−3^/mm^3^	229	19	12	16	71
Internal normalized ratio	0.97	0.91	0.94	NT	1.27
Prothrombin time, s	10.7	9.9	10.2	NT	14
Prothrombin activity, %	104	115	110	NT	71
Partial thromboplastin time, s	26.2	46.7	47.5	NT	Not coagulable
Functional fibrinogen, mg/dL	320	274	268	NT	172
D-dimer, ng/mL	NT	NT	1123	NT	1781
Biochemical parameters					
Aspartate aminotransferase, U/L	20	197	527	961	3,129
Alanine aminotransferase, U/L	9	52	155	269	755
Bilirubin, mg/dL	0.5	0.6	0.5	0.7	0.9
Gamma-glutamyl transferase, U/L	22	229	303	388	545
Alkaline phosphatase, U/L	43	187	289	456	679
Lactate dehydrogenase, U/L	172	1,017	1,188	1,801	3,864
Creatinine, mg/dL	0.83	0.6	0.96	0.8	0.77
Sodium, mmol/L	138	136	134	134	137
Potassium, mmol/L	4.3	5.4	4.5	4.5	4.9
Ionic calcium, mmol/L	1.1	NT	1.15	1.06	1.06
Albumin, g/dL	3.9	NT	2.8	2.4	2.3
Glucose, mg/dL	83	87	97	100	117
Uric acid, mg/dL	NT	NT	NT	3.7	NT
C-reactive protein, mg/dL	0.51	1.77		3.77	NT
Procalcitonin, ng/L	0.17	0.22	0.63	NT	NT
Bicarbonate, mmol/L	23.2	NT	21.6	20.8	20
Lactate, mmol/L	1.1	3	1.4	1.2	2.5
Ferritin, ng/dL	NT	NT	NT	>40,000	NT
Ammonia, μmol/L	NT	NT	NT	NT	60
Spontaneous urine protein, g/L	Neg	1.41	NT	NT	**NT**
Erythrocytes in urine, cells/μg	Neg	50	NT	NT	NT
Vital signs					
Temperature, °C	39.2	38.3	37.1	37.2	37.1
Blood pressure, mm Hg	122/59	116/66	110/65	100/60	90/45
Heart rate, beats/min	110	87	70	76	89
Treatments received, IV					
Physiologic serum	N	Y	Y	Y	Y
Doxycycline	N	Y	Y	Y	Y
Piperacillin–tazobactam	N	Y	Y	Y	Y
Levofloxacin	N	Y	N	N	Y
Platelets, 1 pool	N	Y	N	N	Y
Vitamin K	N	N	N	N	Y
Tranexamic acid	N	Y	N	N	Y
Fresh frozen plasma	N	N	N	N	Y
Methylprednisolone	N	Y	Y	Y	Y

*IV, intravenous; neg, negative; N, not collected/administered that day; NT, not tested, Y, yes, collected/administered that day.

**Figure 1:**
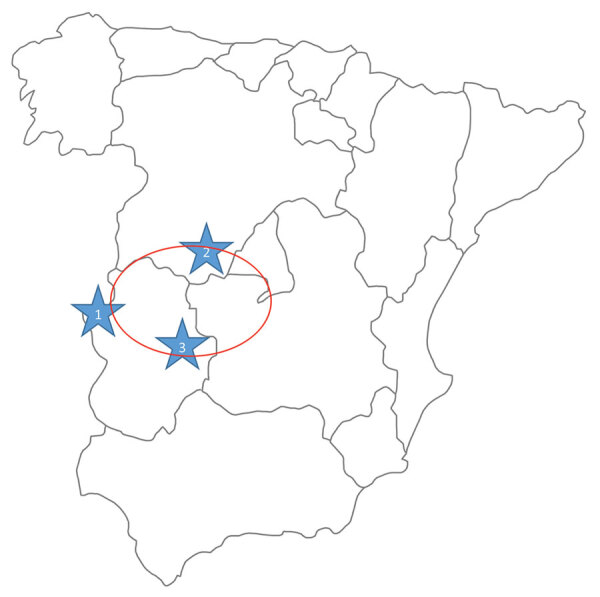
Regions where human infections with Crimean-Congo hemorrhagic fever virus (CCHFV) or infected ticks have been found in Spain. 1, CCHFV hyperendemic focus; 2, human infected by a tick bite in 2016 (Ávila); 3, human infected by a tick bite in 2018 (Badajoz). Red circle indicates area where infected ticks were detected during a surveillance study in 2016.

On August 4, the man returned to the hospital with general discomfort and no improvement. He reported increased stools that he (a retired physician) assumed were associated with the antimicrobial drug and reported probably having been bitten by a tick while participating in boar hunting on July 24 in Helechosa de los Montes (Badajoz, Spain) (Figure 1). The patient had skinned the boar and came into close contact with abundant blood. Physical examination on his return to the hospital was unremarkable, but because of persistent symptoms and the appearance of petechiae and thrombocytopenia, the man was hospitalized for laboratory testing (Table 1) and imaging. 

On August 7, infection with CCHF virus (CCHFV) was considered. The patient progressively worsened and died at the end of the day. On August 8, a blood sample was collected into an EDTA tube and sent to the National Center for Microbiology (Madrid, Spain) for CCHFV diagnostic testing. The sample was inactivated in the Biosafety Level 3 facility by using a QIAamp viral RNA kit (QIAGEN, https://www.qiagen.com) and after addition of ethanol was sent to the Biosafety Level 2 facility. Diagnostic testing was performed by using a RealStar CCHFV RT-PCR Kit 1.0 (Altona Diagnostics, https://altona-diagnostics.com), and CCHFV infection was confirmed by 2 methods: reverse transcription PCR (*1*) (slightly modified to incorporate an internal control for amplification) and a nested reverse transcription PCR (*2*). Results were further confirmed by a World Health Organization Collaborating Center (Public Health England, London, UK).

Molecular and serologic virus detection testing was also performed on additional serum samples. At 6 days after symptom onset, the patient’s viral load (standards kindly provided by Altona Diagnostics) was >10^8^ copies/mL. Specific IgG and IgM against CCHFV were detected by using a commercial indirect immunofluorescence assay (Crimean-Congo Fever Virus Mosaic 2 IFA; Euroimmun, https://www.euroimmun.com), according to the manufacturer’s instructions (Table 2). IgM was detected on day 6 and IgG on day 7, both at very low titers.

**Table 2:** Microbiological test results for Crimean-Congo hemorrhagic fever patient, Spain, August 2018

Result	Days after symptom onset	Viral load, copies/mL	C_t_	IgG			IgM	
				GPC C	N		GPC	N
Serum	6	2,82 10^8^	22	Neg	Neg		Neg	Pos (1/10)
Serum	7	1,54 10^7^	25	Neg	Pos (1/40)		Neg	Pos (1/10)
Blood in EDTA	7	1,58 10^7^	25	NT	NT		NT	NT

*C_t_, cycle threshold; GPC, glycoprotein C; N, nucleoprotein; neg, negative; NT, not tested; pos, positive.

Sequencing of small (S), medium (M), and large (L) segments was performed by using primers previously described (*3*). Complete genome sequences were obtained (GenBank accession nos. MN689738 [S segment], MN689740 [M segment], and MN689741 [L segment]).

**Figure 2:**
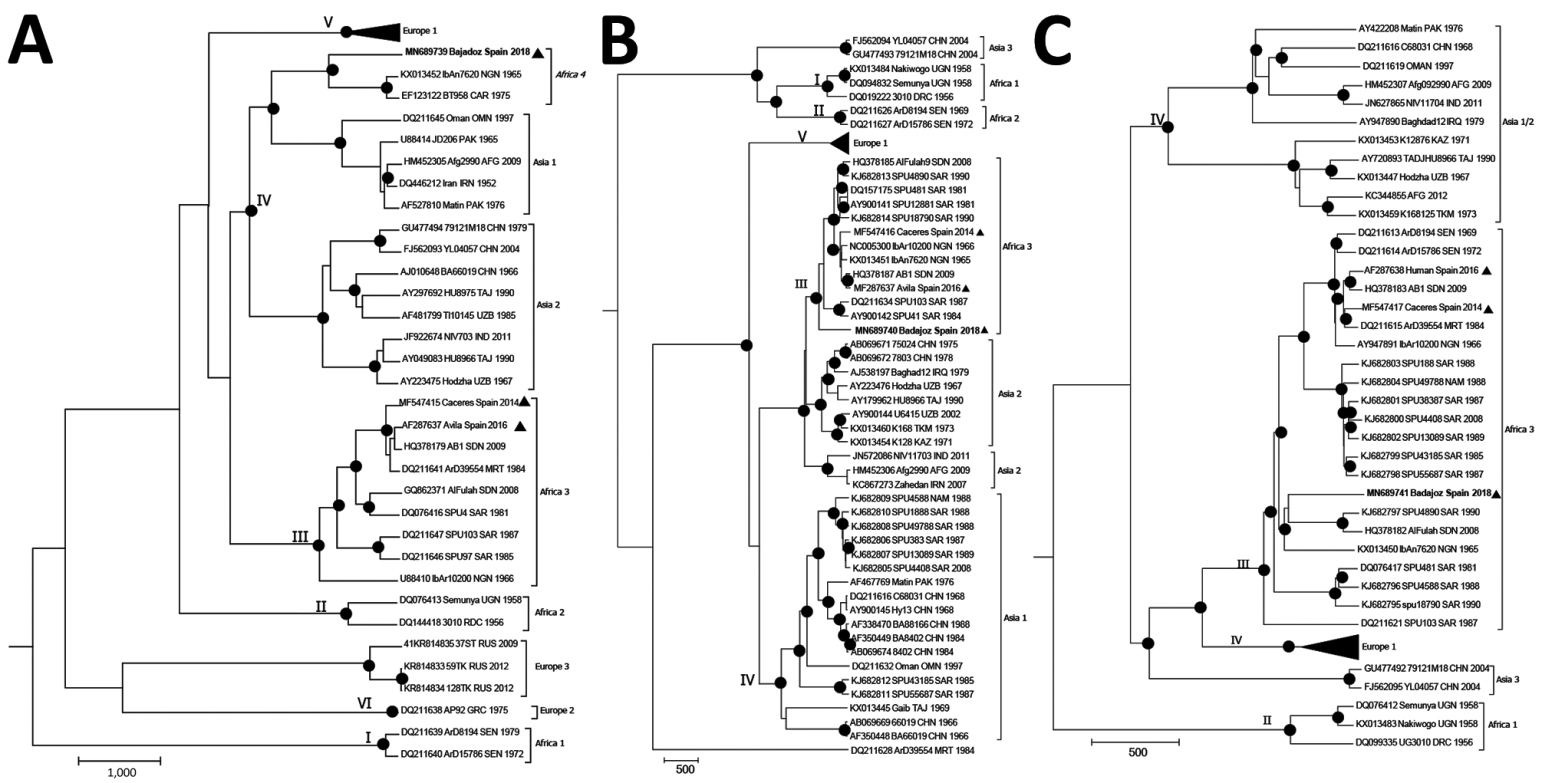
Bayesian phylogenetic trees showing genetic relationships among Crimean-Congo hemorrhagic fever (CCHFV) viruses based on complete small (A), medium (B), and large (C) segment sequences. In the medium segment, the hypervariable mucin-like domain was excluded. We used CIPRES Science gateway (http://www.phylo.org) to implement Bayesian analyses. Black dots indicate nodes with posterior probabilities >0.95; boldface indicates CCHFV strain Badajoz 2018 from Spain; arrowheads indicate other isolates from Spain. Other sequences are named by GenBank accession number, strain, geographic origin, and sampling year. Sequences from this study are included in EMBL/GenBank databases. Roman numerals indicate genotypes, named according to (*4*) with the equivalent clade nomenclature according to (*5*) indicated by brackets: I, West Africa (Africa 1); II, Central Africa (Africa 2); III, South and West Africa (Africa 3); IV, Middle East/Asia, divided in 2 groups corresponding to groups Asia 1 and Asia 2; V, Europe/Turkey (Europe 1); VI, Greece (Europe 2). Italics indicate the proposed new lineage, Africa 4. Scale bars indicate time in years.

Phylogenetic analyses with neighbor-joining and Bayesian (Figure 2) approaches that used MEGA7 (https://www.megasoftware.net) and the Beauti/Beast 1.75 package (https://beast.community) programs show similar results. The strain, Badajoz 2018, belongs to genotype III if the L and M segments are analyzed; however, the S segment is closely related to sequences of genotype IV and shares the highest identity with the strains BT 958 (92.62%) from Central African Republic and IbAn7620 (92.58%) from Nigeria. 

## Conclusions

Phylogenetic analysis of the virus responsible for a fatal case of CCHF in 2018 showed reassortment, indicating a new CCHFV circulating in Spain. The patient was probably infected by a tick bite obtained while hunting. The incubation period for this patient was longer (7 days) than that typical after a tick bite (1–3 days) (*6*); however, the patient also participated in skinning the boar. The geographic location of the hunting site is very close to a natural park, bordering regions where the CCHFV genome has been detected in ticks (*7*) (Figure 1). After returning home, the patient felt ill, but CCHFV was not suspected until 7 days after symptoms appeared, just before he died. No specific treatment was administered. Despite the hospital being located within the region where the first case of CCHFV in Spain was detected, clinician awareness was not high enough to suspect CCHFV infection, partially because the patient diverted attention away from his possible contact with ticks or infected animals. The analytical parameters and the microbiological data are in accordance with described parameters for CCHFV in patients who have died (*8*), although partial thromboplastin time (and not prothrombin time) was altered in the final hemorrhagic phase, in contrast with parameters for the 2016 CCHF patient in Spain (*2*).

Sequence analysis revealed circulation of a CCHFV very different genetically than the one previously described in Spain in humans or ticks (*2*,*3*,*9*,*10*). Complete sequences of viruses detected in humans (2016) and in ticks (2014) have indicated circulation of genotype III viruses, but the virus detected in 2018 is a reassortant in the S segment. Badajoz 2018 L and M segments group within genotype III (with sequences quite different than other sequences from Spain) and the S segment is similar to genotype IV. This S segment is close to the IbAn 7620 strain, isolated from the serum of a goat in 1965 in Nigeria, and the BT 958 strain from the Central African Republic, detected in 1975 and considered by Lukashev et al. (*11*) as an outlier of genotype IV. Genotype IV is formed by 2 genetic lineages, Asia 1 and Asia 2. Because the differences of Badajoz 2018 and related sequences with the Asia strains of genotype IV are remarkable, obtaining more related sequences from Spain or Africa may enable us to split this genotype into other genetic lineages by defining new genetic groups, probably with an origin in Africa. To date, this new genetic lineage contains sequences from 3 geographic regions detected 3 times.

The sequencing results showing 2 virus genetic lineages circulating in Spain indicate that at least 2 introductions have occurred. This situation seems to be distinct from that of the Balkans region, where only 1 virus was introduced from Asia and the virus causing human cases has remained genetically stable for decades (*11*). In fact, circulation of different variants of 1 virus in a small region where it is hyperendemic (Figure 1) in Spain in different years has been described (*10*), showing that the variability of CCHFV in Spain deserves special attention and efforts to get more sequence information.

Because of the high pathogenicity of CCHFV, a detailed medical history of the patient, including travel history and possible risk factors, is crucial for prompt diagnosis to ensure that appropriate infection control measures can be implemented in a timely manner. For the patient that we report, lack of immediate information regarding the tick bite in combination with the nonspecific initial symptoms meant that CCHF was not suspected until day 8 of illness. The public health services performed contact tracing to identify all persons exposed, and none contracted symptomatic CCHF. This case and the description of a new virus do not modify the risk for infection by CCHFV in Spain. Risk is still considered low, although clinicians at hospitals and general practitioners need to be alert to the possibility of new cases.
